# Predictive Biomarkers for the Early Detection of Anastomotic Leaks in Colorectal Surgeries: A Systematic Review

**DOI:** 10.7759/cureus.74616

**Published:** 2024-11-27

**Authors:** Wahidullah Dost, Mohammad Qaher Rasully, Mohammad Nazir Zaman, Wahida Dost, Wahida Ali, Sami A Ayobi, Raisa Dost, Jamaluddin Niazi, Kinza Bakht, Asma Iqbal, Syed Faqeer Hussain Bokhari

**Affiliations:** 1 Surgery/Medicine, Kabul University of Medical Sciences, Kabul, AFG; 2 General Surgery, Jamhuriat Hospital, Kabul, AFG; 3 Cardiac Surgery, Kabul University of Medical Sciences, Kabul, AFG; 4 General Surgery, Kabul University of Medical Sciences, Kabul, AFG; 5 Cardiac Surgery, Kabul university of Medical Sciences, Kabul, AFG; 6 Cardiovascular Surgery, Punjab Institute of Cardiology, Lahore, PAK; 7 Medicine, Sheikh Zayed Medical College and Hospital, Rahim Yar Khan, PAK; 8 Medicine and Surgery, King Edward Medical University, Lahore, PAK; 9 Surgery, King Edward Medical University, Lahore, PAK

**Keywords:** anastomosis, anatomotic leak, colorectal, crp, procalcitonin, surgery, systematic review, wbc

## Abstract

Anastomotic leaks (ALs) remain a serious postoperative complication in colorectal surgery, often resulting in significant morbidity, prolonged hospitalization, and increased mortality risk. This systematic review aims to evaluate the role of predictive biomarkers in the early detection of ALs, focusing on their diagnostic accuracy and clinical utility. Following Preferred Reporting Items for Systematic Reviews and Meta-Analyses (PRISMA) guidelines, a comprehensive literature search was conducted across MEDLINE, Scopus, CENTRAL, and Web of Science, identifying studies that examined biomarkers such as C-reactive protein (CRP), procalcitonin (PCT), and white blood cell (WBC) count in the context of AL. A total of 20 studies met the inclusion criteria, with sample sizes ranging from 59 to 2,655 patients undergoing colorectal surgeries with primary anastomosis. CRP emerged as the most widely studied and reliable biomarker, with studies suggesting that elevated CRP levels, particularly on postoperative days 3-4, can effectively indicate AL risk, showing high negative predictive value. PCT has also shown promise as a complementary biomarker, offering enhanced specificity for infectious complications. Although WBC count alone was a limited predictor, it may add diagnostic value when used with other markers. In addition, innovative biomarkers, such as inflammatory indices in peritoneal fluid, demonstrated potential for further improving AL detection accuracy.

## Introduction and background

Anastomotic leaks (ALs) are among the most severe complications following colorectal surgery, often leading to increased morbidity, prolonged hospitalization, and, in some cases, mortality [1]. The management of ALs presents significant clinical challenges due to the unpredictable nature of their onset and the subtle early symptoms, which can easily be overlooked in standard postoperative monitoring [2]. Consequently, the early detection of ALs has become a focal point for improving outcomes in colorectal surgery, as timely intervention can mitigate severe complications and enhance recovery. Recent advancements in biomedical research have highlighted the potential of predictive biomarkers to signal early signs of anastomotic complications, providing a valuable addition to traditional clinical assessments.

Predictive biomarkers, including inflammatory markers such as C-reactive protein (CRP) and procalcitonin (PCT), have garnered substantial attention due to their measurable fluctuations in response to inflammatory and infectious processes [3]. These markers have demonstrated promise in distinguishing routine postoperative inflammation from the pathological responses indicative of ALs, offering clinicians a more precise and less invasive tool for postoperative monitoring. Among these biomarkers, CRP has emerged as the most widely studied due to its accessibility and reliability in tracking inflammatory states, particularly within the first few postoperative days. PCT, while less routinely used than CRP, has also shown potential as a complementary marker, with studies suggesting that it may offer higher specificity in detecting infectious complications like ALs [4,5].

Despite encouraging evidence, the clinical implementation of biomarkers for AL detection is still evolving. Disparities in study methodologies, including variations in sample size, cutoff values, and the timing of postoperative measurements, have presented challenges in establishing standardized protocols for biomarker-based surveillance. Furthermore, while CRP and PCT are well-recognized, other biomarkers and novel approaches, such as the combination of systemic and peritoneal inflammatory markers, have yet to be fully explored. Understanding the predictive value of these biomarkers, along with the optimal timing and thresholds for their measurement, is essential to developing effective, evidence-based protocols that can improve the early detection of ALs [6].

This systematic review aims to critically evaluate current evidence on the use of predictive biomarkers for the early detection of ALs in colorectal surgeries. By analyzing the sensitivity, specificity, and predictive value of various biomarkers, this review seeks to clarify their role in clinical practice and identify gaps in existing research. In addition, the review assesses emerging trends in biomarker application, such as combined marker models and novel inflammatory indices, and considers the practical implications of biomarker-guided postoperative monitoring. Ultimately, this review aspires to provide a comprehensive understanding of the potential for biomarkers to improve postoperative outcomes in colorectal surgery and highlight areas for future research to optimize AL management and patient care.

## Review

Materials and methods

This systematic review was designed to critically evaluate predictive biomarkers for the early detection of ALs in colorectal surgeries. The review adhered to the PRISMA (Preferred Reporting Items for Systematic Reviews and Meta-Analyses) 2020 guidelines to ensure a high standard of transparency, reproducibility, and methodological rigor [7].

Search Strategy

We performed a comprehensive literature search across MEDLINE (via PubMed), Scopus, Cochrane Central Register of Controlled Trials (CENTRAL), and Web of Science, covering all available publications from inception up to October 2024. The following search terms were used: "(biomarker OR CRP OR procalcitonin OR WBC) AND (anastomotic leak OR AL) AND (colorectal OR rectal OR anastomosis)". We also manually searched the reference lists of key studies and reviews.

Eligibility Criteria

Studies were included if they met criteria based on the population, intervention, comparison, outcomes, and study design (PICO framework). We included studies involving adult patients undergoing colorectal surgery with primary anastomosis and focused on biomarkers (e.g., CRP, procalcitonin, WBC) used to predict ALs. Outcomes of interest included the sensitivity, specificity, and predictive value of biomarkers for early leak detection. Eligible study types were randomized controlled trials (RCTs), observational studies, cohort studies, and case-control studies, while case reports, case series, conference abstracts, gray literature, and non-English language articles were excluded.

Study Selection Process

The study selection was conducted independently by two reviewers who first screened titles and abstracts to exclude studies that did not meet the inclusion criteria. Full-text articles of potentially eligible studies were then retrieved and assessed against the eligibility criteria. Any discrepancies in the selection process were discussed and resolved by consensus with a third reviewer, when necessary.

Data Extraction

Data were extracted using a standardized form by two reviewers independently, covering aspects such as study design, sample size, patient demographics, type and location of surgery, biomarker details (e.g., cutoff values, sensitivity, and specificity), and predictive performance for ALs. Discrepancies were resolved through consensus.

Data Analysis

Due to heterogeneity in biomarker applications, a qualitative synthesis was performed. Findings were summarized and compared to highlight consistencies and discrepancies, with particular attention to factors such as biomarker type, threshold values, and patient populations. Potential influences on predictive performance, such as timing of biomarker measurement and clinical protocols, were also examined.

Results

Study Selection

**Figure 1:**
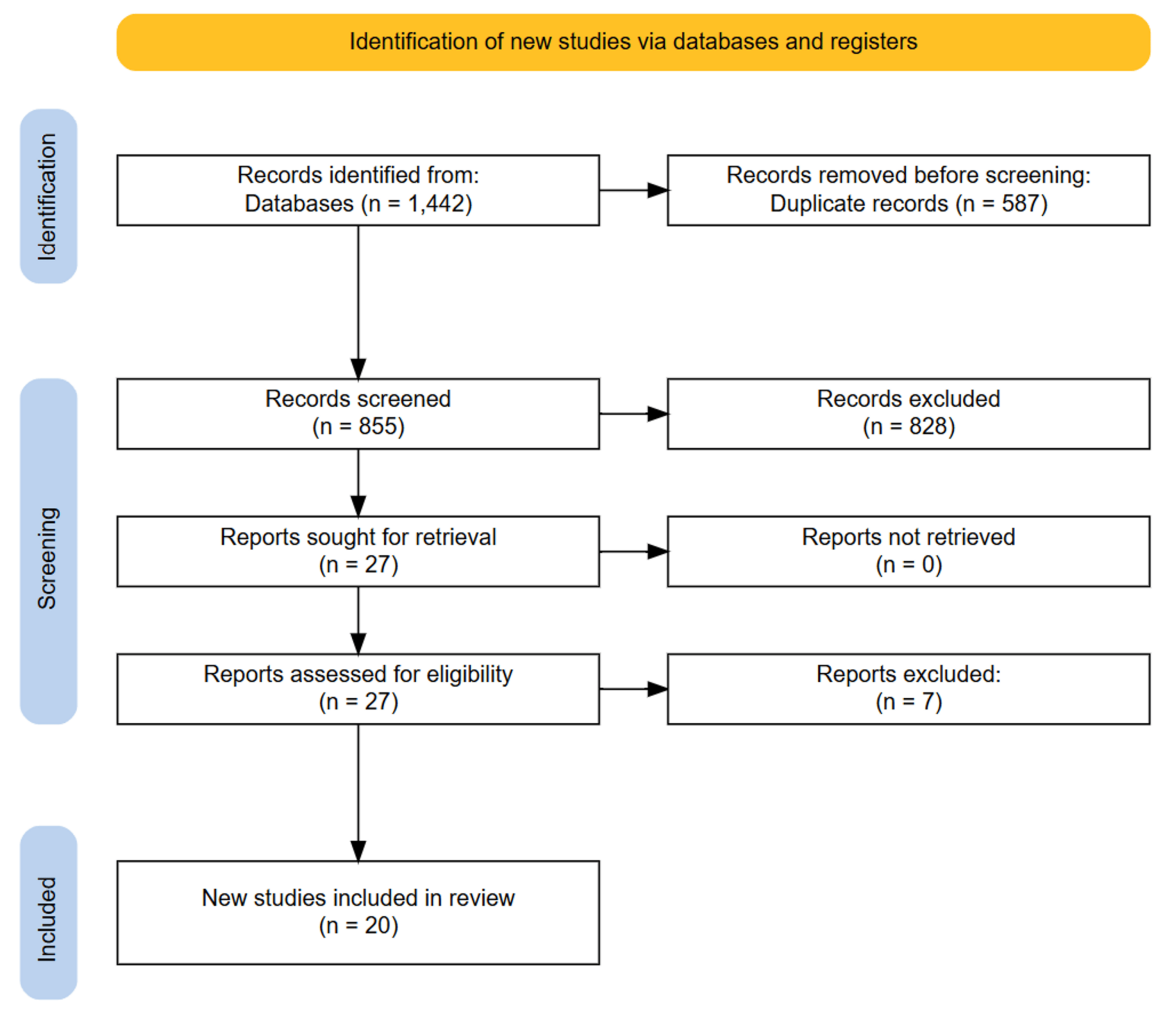
Preferred Reporting Items for Systematic Reviews and Meta-Analyses (PRISMA) diagram illustrating the study selection process.

The initial database search identified 1,442 studies. After removing 587 duplicate entries, the titles and abstracts of 855 studies were screened. Of these, 27 studies were deemed potentially relevant based on the inclusion criteria and underwent full-text review. After a detailed evaluation, 20 studies were included in the final systematic review. A manual search of the reference lists from the selected studies did not reveal any additional eligible studies. The full study selection process is illustrated in the PRISMA flowchart (Figure 1).

Study Characteristics

This systematic review included 20 studies from multiple countries, including Germany, Norway, France, the United Kingdom, Portugal, Italy, India, Egypt, Australia, China, Japan, and Croatia. These studies spanned a variety of study designs, with 12 prospective cohort studies, seven retrospective cohort studies, and one case-control study. Sample sizes ranged from as few as 59 patients to as many as 2,655, providing a broad spectrum of data on patients undergoing colorectal surgery with anastomosis. The study characteristics of the included studies are summarized in the following table (Table 1).

**Table 1:** Study characteristics of the included studies. AL: anastomotic Leak, LAR: low anterior resection, HAR: high anterior resection, NR: not reported

Author	Year	Country	Study design	Sample size	Number of patients with ALs	Number of patients without ALs	Type of surgery	Location of anastomosis
Welsch et al. [8]	2007	Germany	Matched case-control study	383	22 (5.6% in LAR, 7.0% in HAR)	361	Rectal resection with primary anastomosis	Rectal anastomoses
Kørner et al. [9]	2009	Norway	Retrospective cohort	231	18	213	Colorectal resection	Enterocolic, colocolic, pelvic and ostomy
Ortega-Deballon et al. [10]	2010	France	Prospective cohort	133	21	112	Colorectal surgery	Colorectal anastomosis
Platt et al. [11]	2012	UK	Prospective cohort	454	26	428	Colorectal resection	Colorectal anastomosis
Almeida et al. [12]	2012	Portugal	Retrospective cohort	173	24	149	Colorectal surgery with primary anastomosis	Right colon, left colon, rectum, total colectomy
Garcia-Granero et al. [13]	2013	Spain	Prospective cohort	205	17 (11 major, 6 minor)	188	Colorectal surgery (Right colectomy, Left colectomy, Rectal resection, Hartmann reversal, Ostomy closure)	Colorectal anastomosis (Side-to-side, End-to-end, End-to-side, Side-to-end)
Giaccaglia et al. [14]	2014	Italy	Prospective cohort	99	7	92	Elective colorectal surgery (laparotomic or laparoscopic)	Ileo-colic, colo-colic, colo-rectal, or colo-anal
Käser et al. [15]	2014	Germany	Retrospective cohort	1,106	81	1,025	Colorectal surgery	Colonic and rectal anastomoses
Giaccaglia et al. [16]	2016	Italy	Prospective cohort	504	28	476	Colorectal surgery (right colectomy, transverse resection, left colectomy, sigmoid resection, and low anterior resection)	Colorectal anastomosis
Wani et al. [17]	2020	India	Prospective cohort	60	16	44	Colorectal surgery	Colorectal anastomosis
Essa et al. [18]	2021	Egypt	Prospective cohort	130	10	120	Colorectal resection (both open and laparoscopic)	Colo-colic or colorectal anastomosis without covering ileostomy
Rama et al. [19]	2022	Portugal	Prospective cohort	396	25	371	Colorectal resection	Ileocolic, colocolic and near anal verge
Gozalichvili et al. [20]	2022	France	Retrospective cohort	2655	171 (123 included in main analysis)	2484	Elective colorectal surgery	Ileocolic, colo-colic, colorectal (including coloanal and ileoanal)
Zhang et al. [21]	2022	China	Prospective cohort	204	19	185	Laparoscopic anterior resection	Colorectal anastomosis (sigmoidorectostomy)
Qi et al. [22]	2023	China	Prospective cohort	157	7	150	Laparoscopic low anterior resection	Colorectal anastomosis
Isohata et al. [23]	2023	Japan	Retrospective cohort	331	28	303	Colorectal surgery	Sigmoid colon, upper rectum, lower rectum, and other
Choi et al. [24]	2023	Australia	Prospective cohort	361	16	345	Colorectal resection (including right hemicolectomy, transverse colectomy, left hemicolectomy, anterior resection, high/low/ultralow anterior resection, total proctocolectomy)	Colorectal anastomosis
D et al. [25]	2023	Croatia	Prospective cohort	59	8	51	Colorectal resection with primary anastomosis	Right hemicolectomy (n = 23), left hemicolectomy (n =18), low anterior rectal resection (n =18)
Rajabaleyan et al. [26]	2024	UK	Retrospective cohort	1855	57	1798	Hemicolectomy/sigmoid resection	NR
Hu et al. [27]	2024	China	Retrospective cohort	113	11	102	Colorectal resection (36 right colectomies, 18 left colectomies, 9 transverse resections, 23 sigmoid resections, 3 ileocecal resections, 24 low anterior resections)	Colorectal anastomosis

C-reactive Protein (CRP)

CRP emerged as the most extensively studied and reliable biomarker for early AL detection. Multiple studies demonstrated that CRP levels follow a predictable pattern in uncomplicated cases, typically peaking around the postoperative day (POD) 3 and gradually declining thereafter [11-14,18,24,25]. Persistent elevation or secondary rises in CRP levels after POD 3 should raise suspicion for complications, particularly ALs. The studies showed remarkable consistency in identifying POD 3-4 as the optimal timeframe for CRP measurement, with most studies reporting clinically useful cutoff values between 140 and 190 mg/L [8,12,24,26]. Notably, Welsch et al. reported that a CRP cutoff of 140 mg/dl on POD 3 achieved 80% sensitivity and 81% specificity, while Platt et al. found that a cutoff of 190 mg/L on POD 3 yielded similar performance [8,11]. The high negative predictive value (NPV) of CRP is particularly noteworthy, consistently exceeding 95% across multiple studies. This suggests that normal CRP levels in the early postoperative period can reliably rule out AL, potentially facilitating safer early discharge decisions for low-risk patients. However, the relatively lower positive predictive value (PPV) indicates that elevated CRP alone should not be considered diagnostic, but rather as a trigger for more detailed clinical assessment and imaging studies.

Procalcitonin (PCT)

Included studies have highlighted PCT as a promising complementary biomarker to CRP. Garcia-Granero et al. demonstrated that PCT was superior to CRP in predicting major AL, with a cutoff of 0.31 ng/mL on POD 5 achieving 100% sensitivity and 72% specificity [13]. Similarly, Giaccaglia et al. reported that PCT levels below 2.7 ng/mL on POD 3 had excellent specificity and NPV for ruling out ALs [14,16]. The more recent study by Hu et al. (2024) found that combining PCT and CRP measurements on POD 3 significantly enhanced diagnostic accuracy [27]. PCT appears to show greater specificity for infectious complications compared to CRP, potentially helping differentiate ALs from other causes of postoperative inflammation. However, the optimal timing and cutoff values for PCT measurement vary across studies, suggesting a need for standardization before widespread clinical implementation.

White Blood Cell Count (WBC) and Other Traditional Markers

WBC count, despite its routine measurement, demonstrated limited utility as a standalone predictor of ALs. Multiple studies found that WBC values showed no significant differences between the AL and non-AL groups until relatively late in the postoperative period (POD 6-7) [8-12,15,17,18]. However, some studies suggest that combining WBC with other markers, particularly CRP or PCT, may enhance diagnostic accuracy. The study by Käser et al. uniquely identified hyponatremia as a potential marker, with the combination of leukocytosis and hyponatremia achieving moderate sensitivity and specificity [15].

Novel Biomarkers and Combinations

Several studies explored innovative approaches to AL detection. Wani et al. investigated peritoneal fluid biomarkers, finding that local markers (IL-6, IL-10, TNF-α) demonstrated higher sensitivity and specificity compared to systemic markers [17]. Qi et al. developed a promising predictive model combining the CRP/albumin ratio (CAR), systemic immune-inflammation index (SII), and peritoneal IL-6, achieving good discriminative ability [22]. The incorporation of drain fluid analysis and novel inflammatory markers represents an exciting direction for future research [17]. The study by Gozalichvili et al. provided valuable evidence supporting the clinical implementation of biomarker-based protocols. Their analysis demonstrated that implementing a CRP-based screening protocol (triggers: >150 mg/L at POD 3 or >125 mg/L at POD 4) led to earlier detection, increased anastomotic preservation rates, and reduced severity of complications [20]. This real-world validation supports the practical utility of biomarker-based surveillance strategies. The summary of the main findings of the included studies is summarized in the following table (Table 2).

**Table 2:** Summary of the main findings of included studies POD: post-operative day, CRP: C-reactive protein, WBC: white blood cell count, PCT: procalcitonin, PPV: positive predictive value, NPV: negative predictive value, AUC: area under the curve, IL: interleukin, TNF-α: tumor necrosis factor alpha, TLC: total leukocyte count, ECC: eosinophil cell count, CLP: calprotectin, AUROC: area under the receiver operating characteristic, NLR: neutrophil-to-lymphocyte ratio, LMR: lymphocyte-to-monocyte ratio, PLR: platelet-to-lymphocyte ratio, LCR: lymphocyte-to-CRP ratio, PNI: prognostic nutritional index, CAR: CRP/albumin ratio, SII: systemic immune-inflammation index, GLU: glucose, PA1b: prealbumin, IFN-γ: interferon gamma, EWS: early warning score, ROC: receiver operating characteristic, Hmin: minimum height, Hmax: maximum height, Wmin: minimum width, Wmax: maximum width, LEAK: hroup with anastomotic leak, HEAL: group without anastomotic leak (healed)

Author	Type of biomarker(s)	Main findings	Conclusions
Welsch et al. [8]	CRP, WBC, body temperature	CRP peaked on POD 2 (median 140 mg/l) in uncomplicated cases and declined thereafter. In complicated cases, CRP remained elevated after POD 2. The CRP cutoff value of 140 mg/dl on PODs 3 (sensitivity 80.0%, specificity 81.0%, PPV 85.7%) and 4 (sensitivity 54.3%, specificity 92.3%, PPV 90.5%).	WBC and temperature were within normal range in the early postoperative period. Persistent CRP elevation and elevation above 140 mg/dl on PODs 3-4 are predictive of infectious postoperative complications and should prompt an intense clinical search for inflammatory processes, especially ALs if pneumonia and wound infection are excluded.
Kørner et al. [9]	CRP and WBC	Increased CRP on POD 3, with a cutoff of 190 mg/dL (sensitivity 0.82, specificity 0.73, AUC 0.82). Diagnostic accuracy is similar on PODs 5 and 7.	Serial CRP measurements help detect ALs after colorectal resection.
Ortega-Deballon et al. [10]	CRP and WBC	The AUC for CRP was 0.653 on POD 2, 0.716 on POD 4, and 0.845 on POD 6, while the AUC for WBC was 0.561 on POD 2, 0.687 on POD 4, and 0.701 on POD 6. A CRP cutoff of 125 mg/L on POD 4 had a sensitivity of 0.818 and a negative predictive value of 0.958 for detecting AL.	CRP is a promising biomarker for detecting ALs in the postoperative period.
Platt et al. [11]	WBC, albumin, and CRP	CRP levels were significantly higher from POD 3 onward in those who developed an AL compared to those who did not (all p < 0.001). Increased levels of POD 3 were the earliest predictive markers of an AL, with an AUC of 0.84 (p < 0.001) and an optimal cutoff value of 190 mg/L, sensitivity of 0.77, and specificity of 0.80. On POD 4, the AUC for AL was 0.83 (p < 0.001) with an optimal cutoff of 125 mg/L, sensitivity of 0.77, and specificity of 0.76.	CRP measurement on POD 3 is useful for predicting ALs in colorectal cancer surgery patients.
Almeida et al. [12]	CRP and WBC	Daily average values of serum CRP were significantly higher in the leakage group starting at POD 2 (p=0.003). A cut-off value of 140 mg/L on POD 3 had a sensitivity of 78% and specificity of 86%. WBC values showed no significant differences between groups until POD 6.	Early and persistent elevation of CRP after colorectal surgery with anastomosis is a marker of AL. A cut-off value >140 mg/L on POD3 maximizes sensitivity and specificity.
Garcia-Granero et al. [13]	PCT and CRP	1. PCT was a better predictor than CRP for major AL. 2. The best cutoff was PCT at POD 5 (0.31 ng/mL) with 100% sensitivity, 72% specificity, 100% NPV, and 17% PPV. 3. For CRP at POD 5, a cutoff of 135 mg/L had 73% sensitivity, 83% specificity, 98% NPV, 20% PPV. 4. Both PCT and CRP showed AUC >0.800 after POD 3 for major AL.	Both PCT and CRP are useful predictors for severe AL, with a high negative predictive value on PODs 3-5. PCT increase is more reliable than CRP in predicting major AL. The increase in both biomarkers precedes clinical and radiological diagnosis.
Giaccaglia et al. [14]	PCT, CRP, WBC	PCT levels in POD 3 were significantly higher in AL patients (mean 4.97 ng/mL) vs. other complications (2.27 ng/mL) or no complications (1.12 ng/mL) groups (p = 0.007). PCT <5 ng/mL in POD 3 had 96.7% NPV and 95.7% specificity for AL. In POD 5, PCT <2.0 ng/mL had 96.7% NPV and 94.6% specificity.	PCT is an earlier, more sensitive, and reliable marker of AL compared to CRP and WBC. Increased PCT levels in early PODs may provide more effective AL detection before clinical symptoms appear. Normal PCT values might facilitate safe and early discharge.
Käser et al. [15]	Serum sodium levels and WBC count	1. Hyponatremia (<136 mmol/l) was present in 23% of patients with leak vs 15% without (p < 0.001). 2. Mean serum sodium: 138.8 mmol/l in leak group vs. 140.5 mmol/l in non-leak group. 3. Hyponatremia had a specificity of 93% and sensitivity of 23%. 4. Combination of leukocytosis or hyponatremia had sensitivity 68%, specificity 75%, PPV 18%, and NPV 97%.	Hyponatremia could be a specific and relevant marker of AL after colorectal surgery. If hyponatremia and leukocytosis are present after colorectal surgery, AL should be suspected and a CT scan with rectal contrast dye is recommended.
Giaccaglia et al. [16]	PCT, CRP, and WBC	PCT and CRP showed similar AUC in POD 3 (0.775 vs. 0.772). PCT showed better AUC than CRP in POD 5 (0.862 vs 0.806). Both are better than WBC. PCT <2.7 ng/mL in POD 3: specificity 91.7%, NPV 96.9%. PCT <2.3 ng/mL in POD 5: specificity 93%, NPV 98.3%.	Combining PCT and CRP improved AL diagnosis in POD 5. PCT and CRP demonstrated good NPV for AL in both PODs 3 and 5. Low levels of PCT with low CRP appear to be early and reliable markers of AL after colorectal surgery. These biomarkers could be safely added as discharge criteria after colorectal surgery.
Wani et al. [17]	CRP, WBC count, PCT, and peritoneal IL-6, IL-10, and TNF-α	The sensitivity, specificity, PPV, NPV, and accuracy of different markers, respectively, are CRP: 0.538, 0.125, 0.800, 0.040, 0.483; TLC: 0.981, 0.125, 0.879, 0.500, 0.866; PCT: 0.800, 0.040, 0.538, 0.125, 0.483; IL-6: 0.558, 1.000, 1.000, 0.258, 0.616; IL-10: 0.827, 0.750, 0.956, 0.400, 0.816; TNF-α: 0.846, 0.875, 0.978, 0.467, 0.850.	While systemic biomarkers are poor predictors of AL after colorectal surgery, peritoneal fluid drain biomarkers demonstrate significantly higher sensitivity and specificity for this purpose.
Essa et al. [18]	CRP, PCT, WBC	CRP on POD-3: Best cut-off value >30.1 mg/l (90% sensitivity, 100% specificity). WBC on POD-3: Best cut-off value >7.1×109 cell/l (90% sensitivity, 72% specificity). PCT on POD-5: Best cut-off value >1.7 ng/ml (100% sensitivity, 84% specificity).	The analysis of CRP and WBC on POD-3 together with PCT serum concentrations on POD-5 is crucial for the early detection of AL in either open or laparoscopic colorectal resection surgery.
Rama et al. [19]	CRP, WBC, ECC, PCT, and CLP	WBC and ECC showed better predictive effects on POD 5 (AUROC = 0.62 and 0.70) with high NPVs of 0.94-0.98. PCT had a predictive effect on POD 5 (AUROC = 0.61) but low accuracy, although it maintained high NPVs on PODs 3, 4, and 5 (0.96, 0.95, and 0.96, respectively). The mean CRP was significantly higher in AL patients on POD 5 (195.5 ± 139.9 mg/L vs. 59.5 ± 43.4 mg/L; P < 0.00001), with an NPV of 0.98. The mean CLP on POD 3 was also higher in G3 than G1 (5.26 ± 3.58 μg/mL vs 11.52 ± 6.81 μg/mL; P < 0.00005). The combination of CLP and CRP on POD 3 showed high diagnostic accuracy (AUC = 0.82), reducing the time to AL diagnosis by 5.2 days.	The combination of CRP and CLP enhances the diagnostic accuracy for AL, potentially leading to quicker diagnoses.
Gozalichvili et al. [20]	CRP	CRP >150 mg/L at D3 or >125 mg/L at D4 triggered CT scan protocol. In the "after" implementation period (2016-2020) vs. "before" (2010-2013): lower anastomotic takedown (33.3% vs. 52.9%, p = 0.030). Higher anastomosis preservation (68.1% vs. 49%, p = 0.034). Fewer severe complications (56.9% vs. 66.7%, p = 0.017). Earlier CT scan (4 vs. 6 days, p < 0.001). More days out of hospital (9.5 vs. 0 days, p < 0.001).	A CRP-based protocol for screening AL after colorectal surgery was associated with increased anastomotic conservation, decreased impact and severity of leaks, and shorter hospital stays. Early detection through CRP monitoring enabled more timely interventions before clinical symptoms appeared.
Zhang et al. [21]	Anastomotic ring measurements (Hmin, Hmax, Wmin, Wmax), NLR, and CRP	Minimal height (Hmin) of anastomotic rings was lower in the LEAK group vs. the HEAL group (4.4±0.5 mm vs 5.2±0.8 mm; p < 0.001). NLR at POD 5 had an ROC-AUC of 0.93. Hmin had an ROC-AUC of 0.81. The optimal Hmin cut-off was 4.95 mm (sensitivity 56.8%, specificity 84.2%).	AL occurred after a mean of 5.8±1.4 days. The minimal height of anastomotic rings is a good predictor of AL, while postoperatively the NLR was superior to CRP in the prediction of AL. Proper configuration of anastomotic rings is essential to prevent leakage. A low minimal height represents a technical flaw and indicates the risk of leakage with high specificity.
Qi et al. [22]	WBC, NLR, LMR, PLR, CRP, LCR, PNI, CAR, SII, NLR, PCT, GLU , PA1b, and peritoneal IL-1β, IL-6, IL-8, IL-17A, IFN-γ, pH, IL-10, and TNF-α	CAR on POD 1, and SII and peritoneal IL-6 on POD 3, were independent predictors of early symptomatic AL. Optimal cutoffs were CAR: 1.04, SII: 916.99, and IL-6: 26,430.09 pg/ml. A nomogram incorporating these had a c-index of 0.812, suggesting clinical utility.	The combination of CAR, SII, and peritoneal IL-6 may help predict early symptomatic AL.
Isohata et al. [23]	CRP, WBC, and neutrophil ratios	WBC count on POD 7, CRP levels, and neutrophil ratios on PODs 1, 3, and 7 were significantly higher in the AL group (WBC count, p = 0.003; CRP levels, p = 0.008, p < 0.001, p < 0.001; neutrophil ratio, p = 0.010, p < 0.001, p < 0.001). AUCs of 0.67 for WBC count on POD 7, 0.64 for CRP on POD 1, 0.81 on POD 3, and 0.86 on POD 7; neutrophil ratios were 0.64 on POD 1, 0.82 on POD 3, and 0.71 on POD 7. The CRP cut-off was 10.91 mg/dL on POD 3 (sensitivity 0.714, specificity 0.835, PPV 0.290, NPV 0.969) and 4.58 mg/dL on POD 7 (sensitivity 0.821, specificity 0.872, PPV 0.377, NPV 0.981).	Higher WBC counts, CRP levels, and neutrophil ratios in the AL group indicate significant inflammatory responses, with CRP on PODs 3 and 7 showing promising diagnostic utility for identifying complications.
Choi et al. [24]	CRP	CRP values at POD 3 were the best predictor for AL. CRP cutoff levels <182 mg/L on POD3 was a good predictor of no AL (sensitivity 88%, specificity 87%, PPV 28.6%, NPV 99.1%).	Patients with a CRP cutoff value of <182 mg/L at POD 3 may be earmarked for early discharge if clinically appropriate.
D et al. [25]	Serum CRP and intraperitoneal fluid CRP	1. Serum CRP levels below 121 mg/L on POD 4 predicted good anastomotic healing. 2. Serum CRP showed statistically significant changes from POD 2 to POD 4 (p < 0.002). 3. For intraperitoneal CRP, only POD 4 levels were predictive with a cut-off of 55.20 mg/L. 4. Serum CRP had higher sensitivity (85.7%) and specificity (78.0%) compared to intraperitoneal CRP.	1. Serial measurements of serum and intraperitoneal CRP in the first four PODs provide valuable information about anastomotic healing. 2. Serum CRP has higher sensitivity and accuracy in predicting ALs compared to intraperitoneal CRP. 3. No decline in CRP levels after POD 2 should raise suspicion for ALs.
Rajabaleyan et al. [26]	EWS and CRP	From PODs 3 to 7, CRP was >200 mg/L in patients with AL and <200 mg/L in those without (p < 0.05). From POD 1 to 5, EWS was >2 with leakage and <2 without (p < 0.05). On POD 3, the best cutoffs were 2.4 for EWS and 180 mg/L for CRP (AUC 0.89, sensitivity 90%, specificity 70%).	An EWS of 2.4 and CRP of 180 mg/L on POD 3 after colon surgery with anastomosis are threshold values to trigger AL investigation and treatment.
Hu et al. [27]	CRP and PCT	1. On POD 3, the mean CRP was 325.95 mg/L in ALs vs. 131.26 mg/L in non-AL patients. 2. On POD3, the mean PCT was 12.56 ng/mL in AL vs. 2.82 ng/mL in non-AL patients. 3. For CRP on POD 3: cut-off 235.64 mg/L; sensitivity 96%; specificity 89.42%. 4. For PCT on POD3: cut-off 3.94 ng/mL; sensitivity 86%; specificity 93.56%.	Combining PCT and CRP measurements on POD 3 enhances diagnostic accuracy for ALs (AUC: 0.92, sensitivity: 90%, specificity: 100%, cut-off: 0.44).

Discussion

The findings from this systematic review have significant implications for both clinical practice and future research directions in colorectal surgery. The emergence of reliable biomarker patterns, particularly CRP and PCT, represents a paradigm shift in postoperative monitoring strategies for colorectal anastomosis. While traditional clinical assessment remains fundamental, the integration of objective biochemical markers provides an additional layer of surveillance that could potentially transform the standard of care. A key insight from the collective evidence is the potential for biomarker-guided protocols to facilitate more personalized postoperative care pathways. The ability to stratify patients based on their biochemical profiles opens new possibilities for tailored monitoring strategies. For instance, patients demonstrating favorable biomarker trends might benefit from accelerated recovery protocols and earlier discharge, while those showing concerning patterns could receive intensified surveillance and preemptive interventions. This approach aligns with the broader movement toward precision medicine in surgical care.

The economic implications of biomarker-guided protocols warrant careful consideration. While routine biomarker monitoring incurs additional costs, these must be weighed against the potential savings from prevented complications, reduced length of stay, and optimized resource utilization [28]. The high negative predictive value of certain biomarkers suggests potential cost savings through confident early discharge of low-risk patients. However, the implementation of such protocols requires careful consideration of local resources, laboratory capabilities, and healthcare system constraints. The findings also highlight the evolving role of surgeons in interpreting and acting upon biomarker data. Traditional surgical training emphasizes clinical acumen and technical expertise, but the integration of biochemical monitoring requires additional competencies in data interpretation and risk stratification. This suggests a need for updated surgical education curricula that incorporate biomarker-based decision-making and standardized protocols for responding to concerning trends [29].

An important consideration emerging from this review is the potential impact of biomarker monitoring on surgical decision-making and timing of interventions. The ability to detect subclinical anastomotic complications could lead to earlier, less invasive interventions, potentially preserving anastomoses that might otherwise require takedown [30]. However, this also raises questions about the risk of overtreatment and the appropriate threshold for intervention based on biomarker trends alone. The role of emerging technologies and point-of-care testing deserves particular attention. While current biomarker monitoring typically relies on laboratory-based testing, the development of rapid, bedside assays could further revolutionize postoperative monitoring. Such technologies could enable more frequent measurements and real-time trending, potentially improving the precision of early detection strategies. The findings also raise important questions about the interaction between biomarker profiles and various surgical approaches. As minimally invasive techniques continue to evolve, understanding how different surgical approaches affect post-operative inflammatory responses and biomarker patterns becomes increasingly relevant. This knowledge could inform both surgical decision-making and postoperative monitoring strategies.

The potential role of artificial intelligence and machine learning in interpreting biomarker data represents an exciting frontier. The complex patterns of multiple biomarkers, combined with clinical variables and patient characteristics, could be ideal for machine-learning applications [31]. Such tools could potentially provide more accurate risk prediction and help optimize the timing of interventions. An important consideration is the potential impact of biomarker monitoring on patient anxiety and experience. While objective measurements may provide reassurance to some patients, frequent testing and result monitoring could increase anxiety for others. This suggests a need for careful patient education and communication strategies regarding the role and interpretation of biomarker monitoring. Looking forward, the integration of biomarker monitoring into enhanced recovery after surgery (ERAS) protocols represents a promising area for development. The objective data provided by biomarkers could help optimize ERAS pathways and provide more confident decision-making regarding patient progression through recovery milestones. The ethical implications of biomarker-based decision-making also warrant consideration. As these protocols become more widespread, questions arise about the appropriate balance between algorithmic decision-making and clinical judgment, as well as the implications of false positive or negative results. This suggests a need for careful protocol development that incorporates both objective measurements and clinical assessment.

Several limitations of the current evidence base warrant consideration. First, there was significant heterogeneity in study design, surgical procedures, and definition of ALs across studies. Second, the timing of biomarker measurements and cutoff values varied considerably, making direct comparisons challenging. Third, most studies focused on single biomarkers rather than evaluating combinations or sequential measurements, potentially missing opportunities for improved diagnostic accuracy. Additionally, the influence of various confounding factors, such as preoperative radiation, immunosuppression, and minimally invasive versus open surgical approaches, was not consistently addressed across studies. The impact of these variables on biomarker performance requires further investigation.

The validation of biomarker combinations for ALs is essential, as further studies should aim to determine the optimal combination and timing of multiple biomarkers, particularly CRP and PCT, to maximize diagnostic accuracy. In parallel, there is a pressing need for large-scale validation of standardized biomarker-based surveillance protocols. These protocols should include specific trigger values that signal when additional imaging or intervention is warranted. In addition, the development of novel biomarkers, such as peritoneal markers and emerging inflammatory mediators, may offer more precise indicators of anastomotic healing, warranting further investigation. Risk stratification represents another critical area for advancement; creating predictive models that integrate preoperative risk factors, surgical variables, and postoperative biomarker trends could allow for more personalized surveillance strategies. Finally, conducting cost-effectiveness analyses to compare the economic impacts of routine biomarker monitoring versus standard clinical surveillance is vital for informing policy decisions.

## Conclusions

This systematic review confirms the utility of biomarkers, particularly CRP and PCT, in the early detection of ALs following colorectal surgery. The high negative predictive value of these markers can help identify low-risk patients, while elevated levels should prompt increased surveillance and early intervention when appropriate. Routine CRP monitoring should be standard in the early postoperative period, with particular focus on CRP values measured between PODs 3 and 4. CRP levels below the established thresholds (140-190 mg/L) during this timeframe can help identify low-risk patients who may be suitable for early discharge. Conversely, persistent elevation or secondary increases in CRP should prompt immediate clinical assessment and consideration of imaging studies. Where available, PCT measurement could provide added diagnostic value, especially in cases where CRP results are inconclusive. Importantly, biomarker results should always be interpreted alongside clinical findings and should not be the sole criterion for making intervention decisions. Future studies should focus on validating biomarker combinations and developing more personalized surveillance strategies to enhance the early detection and management of this serious surgical complication.
